# The role of miRNAs in COVID-19 disease

**DOI:** 10.2217/fvl-2020-0389

**Published:** 2021-03-24

**Authors:** Mona Fani, Milad Zandi, Saeedeh Ebrahimi, Saber Soltani, Samaneh Abbasi

**Affiliations:** 1 ^1^Department of Pathobiology & Laboratory Sciences, School of Medicine, North Khorasan University of Medical Sciences, Bojnurd, Iran; 2 ^2^Department of Virology, School of Public Health, Tehran University of Medical Sciences, Tehran, Iran; 3 ^3^Research Center for Clinical Virology, Tehran University of Medical Sciences, Tehran, Iran; 4 ^4^Department of Medical Microbiology, Faculty of Medicine Science, Kerman University of Medical Sciences, Kerman, Iran; 5 ^5^Abadan Faculty of Medical Science, Abadan, Iran

**Keywords:** coronavirus, COVID-19, miRNA, SARS-CoV-2

## Abstract

Nowadays, the SARS Coronavirus 2 (SARS-CoV-2) infection is recognized as the primary cause of mortality in humans. SARS-CoV-2 is transmitted through human-to-human contact and is asymptomatic in most patients. In addition to approved vaccines against SARS-CoV-2 infection, miRNAs may also be promising options against this new virus. miRNAs are small and noncoding RNAs 18–25 nucleotides in length that target the mRNAs to degrade them or obstruct their translation miRNAs act as an observer in cells. This study reviewed the literature on the potential role of cellular miRNAs in the SARS-CoV-2-host interplay as a therapeutic option in COVID-19 patients.

Coronavirus disease 2019 (COVID-19) caused by SARS Coronavirus 2 (SARS-CoV-2) has spread fast from China to everywhere around the world since December 2019 [1]. The symptoms of this novel Coronavirus are fever, cough, severe pneumonia, with an incubation period of 1–14 days. Other human coronaviruses (229E, OC43, NL63 and HKU1 strains) cause common cold symptoms in patients [2]. Coronavirus belongs to the Coronaviridae family and Nidovirales order. The subgroups of this family are alpha (α), beta (β), gamma (γ) and delta (δ) coronavirus [3]. After SARS-CoV and the Middle East respiratory syndrome (MERS-CoV) in 2002 and 2012, SARS-CoV-2 is the third known zoonotic coronavirus in the past two decades. SARS-CoV, MERS-CoV and SARS-CoV-2 are the members of the β subgroup [4].

Coronavirus has a positive and ssRNA with a size ranging around 30kbs in length and 5′cap structure and 3′polyA tail. The spike glycoprotein (S), envelope (E), membrane (M) and nucleocapsid (N) are structural proteins in coronaviruses. S protein is cleaved to S1 and S2 subunits. S1 contains the RBD, which can bind to angiotensin-converting enzyme 2 (ACE2). S2 facilitates the SARS-CoV-2 entry into target cells. Indeed, ACE2 is a physiologically related receptor during the COVID-19 disease [2]. Also, SARS-CoV-2 can bind to transmembrane protease serine 2 (TMPRSS2) and glucose-regulated protein 78 (GRP78) [5]. SARS-CoV-2 employs TMPRSS2 as a cellular serine protease for S priming [2].

M protein is an integral membrane protein that promotes viral assembly by increasing the membrane curvature. The E protein is an integral membrane protein that forms virus-like particles to release the virus. N protein as an antagonist interferon signaling can support viral replication. Coronavirus employs the nonstructural proteins for viral replication to block the host immune system [1,2].

Sequence analysis revealed that SARS-CoV-2 is over 80% identical to SARS-CoV. Moreover, the phylogenetic tree of SARS-CoV showed about 90% identity with bat SARS-CoV [6]. This finding supports the theory of novel coronavirus clusterization versus Bat SARS-like coronavirus. Interestingly, SARS-CoV-2 in the ORF1ab and S regions has a unique amino acid sequence from other coronaviruses [7].

More recent studies have shown that SARS-CoV-2 is associated with acute respiratory distress syndrome, acute lung injury, chronic obstructive pulmonary disease (COPD), diabetes and hypertension [8].

## miRNAs & viral infections

All living organisms and several viruses such as HSV, HIV-1, HCV, dengue and influenza can produce miRNAs. miRNAs are short and noncoding RNA 18–25 nucleotides in length [8,9]. Although, most DNA viruses can produce miRNAs, RNA viruses’ miRNA expression is controversial due to their cytoplasmic replication and lack of access to the nuclear miRNA apparatus. In general, the exact mechanistic roles of viral and cellular miRNAs in viral infections are not fully understood. However, cellular miRNA is produced at the early stage of viral infections due to the antiviral reaction [9].

According to the previous studies, the binding of miRNAs on viral RNA has adverse effects on the virus genome. The miRNA has a significant role in gene regulation via binding to a specific region in 3′-untranslated region (3′-UTR) or open reading frame (ORF) to degrade mRNA or block the translation process [9]. The miRNAs can act as autocrine, paracrine and endocrine cellular regulators [10]. The miRNAs have a central role in the pathogenesis of various diseases. miR-125b, miR-138, miR-199a and miR-21 are correlated with increases in plasma cytokine storms such as TNF-α, IL-1β, IL-6, miR-146a, miR-146b and IL-8 in the acute respiratory distress syndrome and COPD. Indeed, reducing these miRNAs expressions emphasizes on a way to improve acute symptoms and distress due to the downregulation of pro-inflammatory cytokines that increase apoptosis protein expression. On the other hand, the expression of these miRNAs can offer promising diagnostic value to SARS-CoV-2 infection [11]. Hence, it is crucial to widely understand the role of the cellular miRNAs and miRNA-mediated gene-silencing during COVID-19 disease as a new option for developing therapeutics.

## miRNAs against COVID-19

**Figure 1.**
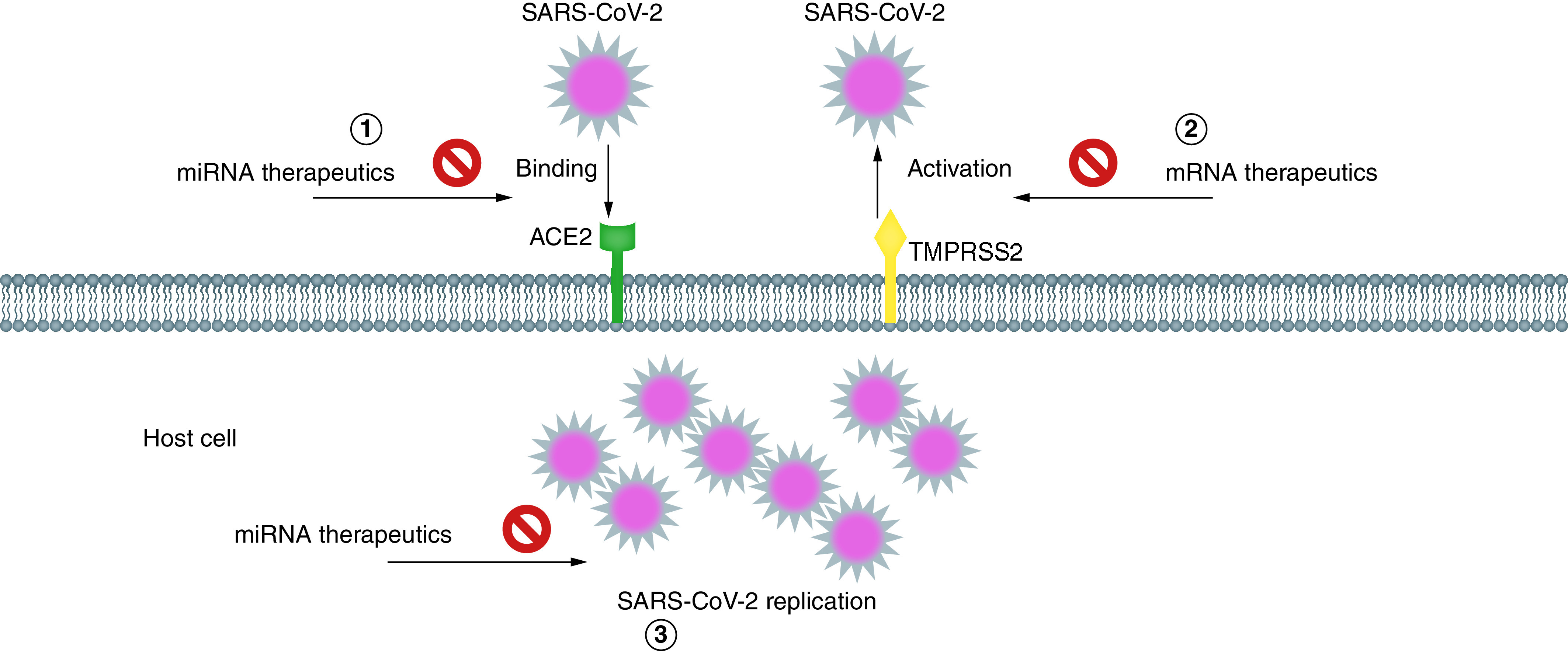
Representation of blocking the binding, activation and replication of SARS-CoV-2 infection by miRNAs.

Generally, there are different ways against the SARS-CoV-2 infection: inhibiting the viral replication, blocking cellular receptors and obstructing the function of viral proteins (Figure 1) [5].

miRNAs can inhibit the viral translation after the attachment of miRNAs to 3′-UTR of the viral genome or target the receptors, structural or nonstructural proteins of SARS-CoV-2 without affecting the expression of human genes.

For instance, ID02510.3p-miRNA, ID00448.3pmiRNA, miRNA 3154, miRNA 7114-5p, miRNA 5197-3p, ID02750.3p-miRNA and ID01851.5p-miRNA, miR-5197-3p [12,13], miR-17-5p and miR-20b-5p [14] mitigate the pathogenesis of COVID-19 disease via binding to the SARS-CoV-2 genome and inhibit its post-transcriptional expression. Nersisyan *et al.* introduced six miRNAs including miR-21-3p, miR-195-5p, miR-16-5p, miR-3065-5p, miR-424-5p and miR-421 that potentially regulated all human coronaviruses through direct binding to the viral genome. The miR-21-3p had the best binding to the human coronavirus genome [15].

Balmeh *et al.* downloaded the nucleotide sequences of 1872 miRNAs from the miRBase database. 42 miRNAs had the highest score, among them miR-1307-3p with the best score showed high affinity to SARS-CoV-2 genome 3′-UTR and were expressed at a high level in comparison with other miRNAs in lung tissue. Indeed, increased expression of miR-1307-3p leads to a reduction in SARS-CoV-2 replication [5]. Also, Chen *et al.* reported that mutations in SARS-CoV-2 3′-UTR lead to virus escape from the host immune system [16]. On the other hand, miR-1307-3p can affect anti-apoptotic proteins like BCL2 to induce apoptosis and inhibit proliferation. In addition, it can inhibit the PI3K pathway to prevent cell cycle proliferation [5]. Moreover, miR-1307-3p involves TGF-β signaling, inflammatory response, oxygen dependency, persistent wheezing and chronic lung diseases [7].

Given that the structural and nonstructural proteins are targeted by human miRNAs, Demirci *et al.* predicted the viral mRNA targets by cellular miRNAs. These proteins are responsible for viral biogenesis, entrance, replication and infection. They found that except for E and ORF6 regions, other viral genes are targeted by multiple cellular miRNAs. For example, miR-203b-3p, in addition to suppressing influenza virus replication, can target ORF1ab and ORF3a SARS-CoV-2. Also, let-7c-5p can target the ORF1ab SARS-CoV-2 and the M1 protein in H1N1 influenza A to inhibit its replication. On the other hand, miR-190a-5p target ORF6 in SARS-CoV-2 and overcome the immune mechanism. Therefore, these miRNAs can be considered as an innate antiviral defense system since SARS-CoV-2 replicates to inhibit the immune system by decreasing the cellular miRNAs. Moreover, Demirci *et al.* reported that miR-148a-3p targets ORF8 in SARS-CoV and prevents viral replication and interspecies transmission. This finding can be another reason for the higher transmissibility of SARS-CoV-2 compared with SARS-CoV [17]. Sardar *et al.* reported six cellular miRNAs that target SARS-CoV-2 proteins: let-7a and miRNA 101 (target the nonstructural proteins), miRNA 126 and miRNA 378 (target the N region), miRNA 23b and (target the S region) [18]. Also, Rad SM *et al.* demonstrated that miR-29b-3p, miR-338-3p, miR-4661-3p, miR-4761-5p and miR-4793-5p may act against the S protein of SARS-CoV-2 [19,20]. Arisan *et al.* reported that miR-8066 could act against the SARS-CoV-2 N gene, which encodes a basic RNA-binding protein that acts as both structural and nonstructural protein. So, targeting this gene can reduce or block the assembly and production of viral particles [7].

Sardar *et al.* showed that considering the importance of cellular receptors, specifically ACE2, in SARS-CoV-2 infection, miRNA 27b regulates the ACE2 receptor [18]. In parallel study, Chauhan *et al.* reported that miRNA 200b-3p, miRNA 200 c-3p and miRNA 429 could act against ACE2 and also let-7c-5p, miRNA 98-5p, let-7 f-5p, let-7a-5p, let-7 g-5p, let-7b-5p, miRNA 4458, let-7e-5p, let-7i-5p, let-7d-5p and miRNA 4500 may regulate the TMPRSS2. Patients with metabolic syndrome, diabetes and cardiac diseases, are prone to SARS-CoV-2 infection due to increased ACE2 receptor expression, so blocking the ACE2 receptor with miRNAs could be a useful therapeutic option to treat COVID-19 [21]. Widiasta *et al.* reported that miR-18 upregulated the ACE2 expression in nephropathy patients and concluded that the anti-miR-18 could be employed for ACE2-related diseases [22]. In another study, Arora *et al.* reported that RIG-I/Ddx58 receptors are highly upregulated in COVID-19 disease. SARS-CoV-2 hijacks the Ddx58 that is involved in miRNA biogenesis and mRNA splicing to promote its replication. Also, miR-124-3p can downregulate the Ddx58 through attachment to 3′-UTR of Ddx58. Therefore, overexpression of miR-124-3p would degrade the Ddx58 and reduce the level of replication of the SARS-CoV-2 genome [23]. Strikingly, SARS-CoV-2 can encode miRNAs to increase overexpression of TMPRSS2 [20] and target several immune signaling such as TLR, IL, TRAF6 signaling and subsequently affect autophagy, mTOR signaling and IFN-I signaling. Moreover, SARS-CoV-2 miRNAs can target genes that are involved in the Ca^+2^ signaling pathway [14].

SARS-CoV-2 is associated with myocarditis, cardiac arrest and acute heart failure, but it is not clear whether these conditions are complications of COVID-19 disease or induced by SARS-CoV-2. The most prominent feature of SARS-CoV-2 infection is the increase in mortality in the elderly and people with underlying conditions. On the other hand, the expression of miRNAs has been reported to be inversely related to age. So, cellular miRNAs easily bind to the viral genome in young people compared with aged individuals and people with underlying conditions. In this regard, Fulzele *et al.* determined several cellular miRNAs against the SARS-CoV-2 genome that are downregulated in the elderly and people with underlying medical conditions. For example, miR-133a (cardiac hypertrophy), miR-1, miR-208, miR-328, miR-21, miR-212 and miR-590 (arrhythmia.) [24], miR-15b-5p (coronary artery disease), miR-15a-5p (kidney disease), miR-520c-3p (obesity/diabetes), miR-30e-3p (myocardial injury), miR-23c(hepatocellular carcinoma), miR-30d-5p (non-small-cell lung cancer), miR-4684-3p (colorectal cancer) and miR-518a-5p (gastrointestinal stromal tumors), are downregulated in pathophysiological condition [25]. Also, in a parallel study it was confirmed that miR-545-3p and miR-519c-3p are associated with COPD and acute exacerbations that often co-occur with respiratory infections [11]. Also, Chow *et al.* found that 128 cellular miRNA can target the SARS-CoV-2 genome. However, most of them have very low or no expression in lung epithelium. Four out of 128 miRNAs, including let-7a-3p, miR-135b-5p, miR-16-2-3p and miR1275, were downregulated. Two out of 128 miRNAs such as miR-155-3p and miR-139-5p were upregulated [26]. Kawasaki disease is associated with COVID-19 disease in children between the ages of 5–15 years. Demongeot *et al.* showed that miR-let-7b is the most upregulated in kawasaki disease. Also, miR-129-5p may have the potential to against the S and ORF10 regions in SARS-CoV-2 infection [27].

A wide range of cytokines are involved in the development of SARS-CoV-2 infection. A study by Arisan *et al.* revealed that miR-8066 elevates the cytokines of PRLR, CXCL6, IL6 and IL17. MiR-5197-3p was known as the most effective therapeutic option due to interaction with the guide RNA of SARS-CoV-2. miR-3934-3p can downregulate TGFBR1 and SMAD3 pathways that are critical for lung fibrosis [7]. Interestingly, due to vitamin D and B3 deficiency in SARS-CoV-2 infection, miR-3934-3p can be associated with vitamin digestion and absorption. The level of IL-10 as one of the pro-inflammatory effector cytokines increased in COVID-19. Nepotchatykh *et al.* demonstrated that hsa-miR-127-3p could regulate the expression of the BCL6 gene and subsequently inhibit the expression of IL-10. This cytokine has anti-inflammatory properties and plays a central role in limiting host immune responses to pathogens [28].

## Conclusion

Due to the importance of miRNAs in infectious diseases and the concerns about the increase in the mortality rate caused by COVID-19 disease, especially in immunosuppressed patients, we reviewed the literature on the potential role of cellular miRNAs in the SARS-CoV-2 and introduced them as therapeutic options for SARS-COV-2 infection.

## Future perspective

In medicine, miRNA can be considered as a novel and attractive biomarker for therapeutic targets. On the other hand, it has been reported that plasma from SARS-CoV-2 infection recovering individuals can be used to treat patients with COVID-19 due to the presence of antivirus miRNAs and antibodies. It is estimated that the miRNAs involved in blocking the ACE2 or TMPRSS2 can regulate the cytokine storm in SARS-CoV-2 infection.

As mentioned above, miRNAs interfere in various biological processes and the heart and lung disease caused by COVID-19 disease. Therefore, by discovering this relationship, scientists and researchers can target miRNA-interaction genes to treat COVID-19 disease. On the other hand, SARS-CoV-2 miRNAs can target the insulin signaling pathway and heart development-related pathways. Therefore they are other reasons for the high mortality rate of COVID-19 disease in underlying and immunosuppressed persons. Several neurological symptoms, including headache, vomiting and nausea, may be associated with SARS-CoV-2 encoded miRNAs that target genes related to brain growth. HIF-1 signaling has a crucial role in cellular maintenance and survival during hypoxic status, but this pathway is deregulated by SARS-CoV-2 miRNAs, resulting in severe consequences in patients with COVID-19 [14]. The gastrointestinal infection in COVID-19 patients is due to the targeting of TMPRSS2 by MR147-3p in the gut [20]. Finally, the comparison of SARS-CoV-2 miRNA with other human coronaviruses showed the role of miRNAs in the clinical characteristics of COVID-19 disease.

Li *et al.* reported that miR-618 is 1.62-fold greater expressed in COVID-19 patients than in healthy persons; on the other hand, miR-618 is related to the downregulation of the immune system. Thus, miR-618 can be a promising target to treat COVID-19 patients [29]. Although miRNA can be an excellent therapeutic option, miRNA degradation and nonspecific targets are its delivery limitations. However, using nanotechnology-based methods and conducting *in vitro* and* in vivo* experiments on animal models can select the best therapeutic target to exert therapeutic effects of miRNAs. Indeed developing nanoformulations of the COVID-19-related miRNAs can successfully transfer the miRNAs to the cells. Also, miRNAs-based therapeutics could be used in the nanovaccines that are specific with minimal off-target effects. Furthermore, nanobased miRNAs vaccines can be used as nasal spray or drops. In the case of COVID-19 disease, nanovaccine in the form of nasal spray seems to be more effective due to the activation of the immune response in the respiratory tract as the common initial site for SARS-CoV-2 virus entry.

**Executive summary**

- miRNAs can inhibit the SARS-CoV-2 infection in different ways: blocking the viral replication, cellular receptors and the function of viral proteins.
- miRNAs interfere in various biological processes and also the heart and lung disease caused by COVID-19 disease.
- miRNA can be considered as a novel and attractive biomarker for the treatment of SARS-CoV-2 infection.
- miRNAs-based therapeutics could be used in the nanovaccines that are specific with minimal off-target effects.
